# Topical application of cellulose membrane for the treatment of non-healing venous leg ulcers

**DOI:** 10.3389/fbioe.2026.1789782

**Published:** 2026-04-21

**Authors:** Ewa Rojczyk, Kinga Spyrka, Andrzej Ślęzak, Marek Kucharzewski

**Affiliations:** 1 Wladyslaw Bieganski Collegium Medicum, Jan Długosz University in Częstochowa, Częstochowa, Poland; 2 Faculty of Medicine, Academy of Silesia in Katowice, Katowice, Poland; 3 Department of General Surgery, Surgical Outpatient Clinic of Healthcare Centre of Jan Paweł II District Hospital in Włoszczowa, Włoszczowa, Poland

**Keywords:** cellulose, dressing, healing, venous leg ulcer, wound

## Abstract

**Introduction:**

Venous leg ulcer (VLU) is a common, chronic condition that may lead to disability. Its frequency increases with age and generates high costs for healthcare systems. Bacterial cellulose is recognized as a natural wound repair material that meets the requirements of modern wound dressings, owing to its superior physicochemical characteristics and favorable biological properties. The aim of this study is to assess the effect of topical application of a cellulose membrane on the healing of chronic VLUs.

**Methods:**

Analysis included 100 patients with chronic VLUs divided into two groups of 50 patients each. Experimental group received a cellulose membrane, whereas control group received a standard antimicrobial calcium alginate dressing with silver. Planimetric measurements of the ulcer area before treatment and then every 7 days were performed, until the ulcer healed completely. The effectiveness of both treatments was compared regarding complete healing time and ulceration area reduction over time.

**Results:**

Patients in experimental group demonstrated significantly shorter complete healing time and a greater reduction in ulcer area over time in comparison to control group. However, in the control group, a temporary acceleration of healing process was observed between 5^th^ and 7^th^ week of treatment.

**Discussion:**

Our findings suggest that cellulose-based biomaterials may represent an effective and biocompatible alternative in the management of VLUs, offering improved healing outcomes and potential benefits for both patients and healthcare systems.

## 1 Introduction

Venous leg ulcers (VLUs) are a common manifestation of chronic venous disease and represent a significant clinical and socioeconomic burden worldwide. They develop as a consequence of sustained venous hypertension, which impairs microcirculation, promotes inflammation, and leads to progressive tissue breakdown. The underlying pathophysiology involves a complex interplay of venous reflux or obstruction, endothelial dysfunction, leukocyte activation, and extracellular matrix degradation, all contributing to delayed healing (Raffetto et al., 2020; Bohn, 2023). Epidemiological data indicate that VLUs account for approximately 60%–80% of all leg ulcers and affect about 1%–2% of the general population, with prevalence increasing with age to up to 4% in individuals over 65 years (Raffetto et al., 2020; Bohn, 2023; Probst et al., 2023). A recent meta-analysis reported a pooled prevalence of 0.32% and an incidence of 0.17%, although substantial heterogeneity between studies has been observed (Probst et al., 2023). VLUs are typically chronic, recurrent, and difficult to manage, often requiring long-term treatment and multidisciplinary care, with high recurrence rates reflecting the persistent nature of the underlying venous pathology (Raffetto et al., 2020; Bohn, 2023). In Poland, as in other industrialized countries, chronic venous disorders are widespread affecting up to half of adults (about 51% of women and 38.3% of men) (Szkiler et al., 2024).

The treatment of VLUs imposes a heavy burden not only on patients and families but also on healthcare systems. In Europe, VLU care consumes an estimated 1%–2% of healthcare budgets. Annual expenditures exceed $3 billion in Australia and about £941 million in the UK. Data from German wound-care centers report costs of roughly €10 000 per patient, with hospitalizations, nursing, and dressings as the main cost drivers. The European Wound Management Association (EWMA) estimates the cost of treating one patient at €6650–10 000 (Purwins et al., 2010; Ma et al., 2014; Guest et al., 2018; de Oliveira et al., n.d.). In Poland, over 600,000 people suffer from VLUs; treatment costs reach about 2% of the healthcare budget (nearly PLN 4 million). The average ulcer duration is 3–4 months, often causing financial strain for both hospitals and patients. National Health Fund data from 2015 (unpublished) estimated wound care costs at 2.15% of gross domestic product (GDP) (Szkiler et al., 2024).

Wound healing involves complex biochemical and physical processes, including the action of local bioactive mediators, increased tensile strength, and changes in skin elasticity. The phases of healing overlap dynamically. The initial hemostatic phase (up to about 3 h post-injury) begins with vascular damage and bleeding, followed by catecholamine- and thromboxane A_2_–induced vasoconstriction. von Willebrand factor, vitronectin, fibronectin, and laminin then trigger the coagulation cascade. In the inflammatory phase (3–24 h after injury), platelets release signaling molecules that attract immune cells such as neutrophils, monocytes, and lymphocytes, promoting clot formation and establishing a provisional matrix for migrating cells. Thrombin converts fibrinogen to fibrin, stabilizing the clot, while mediators from endothelial and mast cells increase vascular permeability. Neutrophils and macrophages cleanse the wound and release additional regulatory factors that coordinate the subsequent stages of healing (Bonnefoy and Legrand, 2000; Broughton and Rohrich, 2005; Skórkowska-Telichowska et al., 2009; Potempa et al., 2014; Targoński, 2015; Wilemska-Kucharzewska et al., 2015; Crawford et al., 2017; Bohn, 2023; Szkiler et al., 2024).

During the proliferative phase (up to ∼14 days), re-epithelialization, granulation tissue formation, and angiogenesis occur. Keratinocytes migrate and proliferate in response to locally produced mediators (Bonnefoy and Legrand, 2000; Skórkowska-Telichowska et al., 2009), while fibroblasts synthesize extracellular matrix (ECM) components such as glycosaminoglycans, proteoglycans, collagen, and elastin, leading to restoration of the epidermal barrier (Bonnefoy and Legrand, 2000; Nowak and Olejek, 2004; Broughton and Rohrich, 2005; Skórkowska-Telichowska et al., 2009; Kahle et al., 2011; Bartoszewicz and Junka, 2012; Bumpus and Maier, 2013; Martin and Nunan, 2015; Rojczyk-Gołębiewska et al., 2015; Targoński, 2015; Wilemska-Kucharzewska et al., 2015; Rojczyk et al., 2016; Han and Ceilley, 2017; Raffetto et al., 2020). In the remodeling phase, fibroblasts differentiate into contractile myofibroblasts that reduce the wound area by 20%–30%. Enzymes such as metalloproteinases and serine proteases remodel the ECM as granulation tissue transitions into a mature scar. After about 1 month, tensile strength reaches approximately 40%, and after 1 year, about 70% of that of normal tissue (Broughton and Rohrich, 2005; Kucharzewski et al., 2007; Misztal-Knyra et al., 2007; Kahle et al., 2011; Martin and Nunan, 2015; Szkiler et al., 2024).

A wound that fails to heal within 4–12 weeks despite appropriate care is considered chronic. In such wounds, the physiological cascade is typically derailed during the inflammatory phase, disrupting subsequent stages. Bacterial burden commonly increases, producing exudate that contains cytokines, growth factors, and proteolytic enzymes. This exudate can inactivate growth factors and degrade the extracellular matrix (ECM), preventing keratinocyte migration and re-epithelialization (Bumpus and Maier, 2013; O’Donnell and Passman, 2014; Dowsett et al., 2021; Szkiler et al., 2024). Although proteases released within the wound normally help clear necrotic tissue and transiently remodel ECM, their expression and activity are tightly regulated under healthy conditions. However, when healing is impaired, excess protease activity leads to uncontrolled ECM destruction, which is a key barrier to closure of chronic wounds (Kucharzewski, 2005; Kahle et al., 2011; Bartoszewicz and Junka, 2012; Martin and Nunan, 2015; Raffetto et al., 2020).

The prolonged healing course can be further complicated by bacterial or fungal infection. Microorganisms may originate from the patient’s own flora or the environment, arriving via passive deposition (air, water) or active transfer (e.g., hands, soil). Rapid microbial proliferation beyond a critical colonization threshold promotes the development of a biofilm, which further sustains inflammation and impedes healing (Kucharzewski, 2005; Kahle et al., 2011; Bartoszewicz and Junka, 2012; Gottrup et al., 2013; Metcalf et al., 2016; Bartoszewicz et al., 2019; Raffetto et al., 2020).

Local treatment of chronic ulcers focuses on re-establishing the balance of factors that drive wound healing. Dressing selection should consider the ulcer’s location and characteristics, depth and extent of tissue loss, exudate volume, presence of infection or biofilm, healing phase, and the condition of the periwound skin (Kahle et al., 2011; Martin and Nunan, 2015; Urbanek et al., 2015; Han and Ceilley, 2017; Raffetto et al., 2020; Szkiler et al., 2024).

The characteristics of an ideal wound dressing were first outlined by Turner in 1979. According to this concept, an optimal dressing (also for chronic wounds) should maintain a moist environment between the wound surface and the dressing while preventing excessive loss of body fluids. It should actively support the healing process and allow for easy, painless dressing changes without causing tissue damage. Furthermore, an ideal dressing should effectively remove excess exudate and toxic substances, remain non-adherent to the wound and provide a barrier impermeable to bacteria. It must also ensure adequate gas exchange, maintain appropriate temperature and pH within the wound bed, and be both non-toxic and non-allergenic (Turner, 1979).

Scientists are striving to create a dressing that meets as many of the above-mentioned criteria as possible. Therefore, atypical dressings, such as those based on bacterial cellulose, are becoming increasingly popular, offering interesting alternatives to traditional ones.

Cellulose is a natural, linear structural biopolymer of anhydroglucose units linked by β(1→4) glycosidic bonds, with the empirical formula (C_6_H_10_O_5_)_n_. The degree of polymerization *n* typically ranges from about 5000 to 10000, depending on the cellulose source. Bacterial cellulose (BC) is rich in hydroxyl groups and glycosidic linkages. The abundance of surface–OH groups confers strong hydrophilicity, giving BC exceptional water-absorption capacity—it absorbs 60–700 times more water than its dry weight. BC qualifies as a nanomaterial: its fibers are smaller than 130 nm in diameter, and the constituent nanofibrils have cross-sections of roughly 5–10 nm × 30–35 nm. It forms a semi-transparent, porous membrane that is selectively permeable to gases (e.g., O_2_, N_2_, CO_2_) yet impermeable to microorganisms. Typical properties include a surface areal density of 9–20 g/m and a near-neutral pH (6–7). The dry film thickness is about 20 μm, increasing to about 50 µm when hydrated. Physicochemical properties BC membrane have been presented in many studies (Ślęzak et al., 2004; Portela et al., 2019; He et al., 2021; Lahiri et al., 2021; Pharasi, 2023; Girard et al., 2024).

Bacterial cellulose was first described in 1886 by A.J. Brown, who identified cellulose production by the acetic acid bacterium *Acetobacter xylinum* (Brown, 1987; Jacek et al., 2019). The first commercial BC-based medical product was Biofill® - a thin film containing 8.5% water (Anton-Sales et al., 2019). This material was used as a temporary skin substitute and wound dressing for basal cell carcinoma, severe burns, abrasions, chronic ulcers, and donor or graft sites. In 1984, Luiz Fernando Xavier Farah discovered that *Acetobacter* species could produce cellulosic membranes suitable as skin substitutes, leading to the establishment of the company Biofill Produtos Bioetecnológicos (Jonas and Farah, 1998).

The first clinical trial using the BC-based Biofill® dressing (produced by *Acetobacter)* was conducted in Brazil in 1986 by Fontana et al. (1990). Around the same time, Gengiflex (a BC membrane for dental applications) was also introduced. Until the early 21st century, Biofill Produtos Bioetecnológicos remained the only company commercializing bacterial cellulose membranes as medical devices. Subsequently, Xylos Corporation developed the BC-based skin substitute XCell, which was later acquired and rebranded by Lohmann & Rauscher as Suprasorb X and Suprasorb X + PHMB (Frankel et al., 2004).

Currently, several commercial bacterial cellulose dressings are available, including Biofill, Gengiflex, XCell, Bioprocess, Dermafill, and Epiprotect. Differences in manufacturing technologies result in slight variations in their physicochemical characteristics (Picheth et al., 2017). Nevertheless, these products have proven effective in the treatment of both acute and chronic wounds. Indeed, in recent years, BC has emerged as a promising biomaterial for advanced wound care. It exhibits a unique, three-dimensional nanofibrous structure, high purity, exceptional water-retention capacity, and mechanical stability. These properties allow BC to closely mimic the native ECM, providing a moist and oxygen-permeable environment conducive to tissue regeneration (Utoiu et al., 2024; Dogaru et al., 2025). Moreover, BC is biodegradable, biocompatible, easily sterilized and has ability to support cell proliferation, which makes it suitable for clinical application (He et al., 2021; Amorim et al., 2022; Horue et al., 2023).

Previous studies have reported favorable outcomes in chronic wounds and venous ulcers treated with BC-based dressings, demonstrating accelerated epithelialization and reduced exudation compared to conventional therapies (Cavalcanti et al., 2017; Silva et al., 2021). Recent preclinical and clinical studies have also shown that BC-based dressings can accelerate wound closure, reduce pain, and are well tolerated by patients (He et al., 2021; Zahel et al., 2022; Horue et al., 2023; Maia et al., 2025). Importantly, the use of BC dressings shortens hospitalization, and ultimately reduces healthcare costs. The reduced need for frequent dressing changes further underscores their cost-effectiveness. Schmitz et al. (2014) demonstrated that BC dressings reduced treatment costs by 60%–70% compared with conventional wound dressings. Moreover, recent advances in BC composites further underline its therapeutic potential for infected and non-healing chronic wounds (Meng et al., 2023; Hou et al., 2024). Advances in biomaterial engineering have also enabled the development of BC-based composite dressings incorporating antimicrobial or bioactive agents, further enhancing their therapeutic potential (Amalia et al., 2024).

Nevertheless, the number of large-scale, randomized clinical trials evaluating BC in chronic VLUs remains limited, and comparative data versus standard antimicrobial dressings are scarce. This gap highlights the need for further clinical evidence to validate the therapeutic potential of BC in chronic ulcer management. Therefore, the aim of this research was to evaluate the clinical effectiveness of a BC membrane in the treatment of chronic VLUs, compared with a standard antimicrobial calcium alginate dressing containing silver. It addresses the current research gap by providing robust comparative clinical data on BC membranes in VLUs, involving a larger patient cohort and objective, planimetric wound assessment until complete healing. By providing clinical evidence on its impact on healing time and ulcer area reduction, this study seeks to address a critical gap in knowledge and support the development of innovative, nature-derived wound management solutions.

Chronic VLUs are notoriously difficult to heal using standard dressings such as hydrocolloids, alginates, or silver-based products. While these materials primarily act as passive barriers that absorb exudate or exert antimicrobial effects, they rarely contribute to the biological repair processes essential for durable healing (He et al., 2021). BC, in contrast, offers both physical protection and biological functionality. Its nanofibrillar network supports fibroblast attachment and proliferation, promotes angiogenesis, and facilitates collagen synthesis (Han et al., 2023; Dogaru et al., 2025). Additionally, BC maintains a stable moist environment, ensures atraumatic dressing removal, and exhibits minimal cytotoxicity compared to silver-based formulations, which may delay epithelialization (Silva et al., 2021; He et al., 2022; Sung et al., 2025).

Thus, BC was selected for the study due to its unique structural and biological properties—its nanofibrous architecture mimics the ECM, promoting fibroblast proliferation, angiogenesis, and collagen deposition (Han et al., 2023; Utoiu et al., 2024; Dogaru et al., 2025). Unlike traditional silver- or alginate-based dressings that mainly address infection control, BC acts as an active healing matrix, supporting tissue regeneration while maintaining an optimal wound microenvironment (Han et al., 2023; Sung et al., 2025). Moreover, BC is a natural, biocompatible, and cost-effective material that minimizes discomfort during dressing changes and reduces inflammatory reactions (Silva et al., 2021; Han et al., 2023).

## 2 Materials and methods

### 2.1 Research group

Our research included 100 patients with chronic venous ulceration of lower limbs, who were randomly divided to two groups (50 patients each) – experimental group treated with bacterial cellulose and control group treated traditionally with a silver-containing dressing. All patients belonged to the same cultural region (central Poland) and to the same ethnic group.

Group allocation was performed using a sealed-envelope randomization procedure. Each participant drew an opaque, sealed envelope that indicated assignment to either the intervention or control group, thereby maintaining participant blinding with respect to group allocation.

Investigation was carried out in the Surgical Outpatient Clinic of the John Paul II District Hospital in Włoszczowa in the period from October 2023 to September 2025. After reviewing the research protocol, the patients gave their informed consent to participate in the study. The study was approved by the Ethics Committee of Medical University of Silesia (protocol code NN-013-133/02 from the 29th of May 2002) and was conducted in accordance with the Declaration of Helsinki.

Each ulcer was diagnosed as a VLU using Doppler sonography. The patients were chosen at random from among those who applied for consultation by surgery doctors. All patients had previously received treatment from a general practitioner, which included elastic compression therapy with bandages or stockings, antiseptic wound irrigation, and the local application of conventional dressings such as hydrogels and hydrocolloids. However, none of these interventions resulted in complete wound healing during the pre-randomization period.

Before research procedure, each ulcer was classified by wound morphology, severity, and location. A systematic description of wound and limb appearance was recorded, including edema, erythema, exudation, granulation, and the presence of fibrin or eschar.

On the basis of clinical and sonographic results, the chronic venous disease of each patient was classified according to CEAP (C–clinical, E–etiological, A–anatomical, P–pathophysiological) classification (Table 1). All wounds showed moderate or heavy exudate.

**TABLE 1:** Classification of venous dysfunction (according to the CEAP classification).

CEAP class	Experimental group	Control group
C6, Ep, As3, Ap18, P_r_	10	11
C6, Ep, As3, Ad13, Ap18, P_r_	8	9
C6, Ep, As2,3, Ad18, P_r_	7	8
C6, Es, As3, Ap18, P_r_	8	7
C6, Es, As3, Ad13, Ap18, P_r_	10	7
C6, Es, As2,3, Ad13, Ap18, P_r_	7	8

C6 – active leg ulcer; Ep–primary lesions; Es–secondary lesions; As–lesions affecting the superficial venous system; Ad–lesions affecting the deep venous system; Ap–lesions affecting perforating veins; P_r_–reflux (axial or perforating veins). Numbers following the anatomical descriptors indicate specific venous segments according to the CEAP, classification of venous segments.

Ankle-brachial index (ABI) was also determined, which was within normal ranges (0.69 to 1,12) for all study participants.

Body mass index (BMI) was determined for all patients using the following formula:

BMI = m/h^2^ [kg/m^2^] where: m is patient’s weight [kg]; h is patient’s height [m]

Exceeding 30 [kg/m^2^] classified the patient as obese.

Importantly, there were no statistical differences between groups regarding anthropometric and clinical data presented in Table 2.

**TABLE 2:** Basic anthropometric and clinical features of patients.

Parameter	Experimental group	Control group
Gender		
Females	35	38
Males	15	12
Age [years]	59.6 (50–79)	60.1 (51–77)
Patient’s living status		
Living alone	15	13
Not living alone	35	37
Patient’s mobility	​	​
Full	40	41
Limited	10	9
Wounding time [months]	23 (17–33)	22 (18–31)
Ulcer area [cm^2^] at the beginning of treatment	14.9 (6.8–23.2)	14.7 (6.6–23.9)
Ulcer location Left crus	36	37 13
Right crus	14	
Ankle-brachial index (ABI)	0.86 (0.7–1.12)	0.89 (0.69–1.11)
Body mass index (BMI)	30.8 (24.1–38.8)	31.1 (24.8–40.2)

Parameters are presented as number of patients or mean value (range).

### 2.2 Research procedures

#### 2.2.1 Pharmacological management and planimetric measurement

All patients received outpatient care and were evaluated weekly by a physician until complete ulcer healing was achieved. Pharmacological management followed a standardized regimen in both groups. Each patient was administered a micronized flavonoid fraction (450 mg diosmin and 50 mg hesperidin), taken as two 500 mg tablets once daily.

All subjects underwent planimetric measurement of the ulcer area before treatment and then every 7 days until the ulcer healed. The procedure was performed as follows: first, homothetic congruent projections of the ulcers were traced onto transparent foil, followed by planimetric measurements of the wound areas using a Mutoh Kurta XGT-1218A3 digitizer (Mutoh, Phoenix, AZ, United States)

For each patient, the rate of ulcer area reduction (*v*_*s*_) per 1 day was calculated according to formula:

$$v_{s}=\frac{S_{i-1}-S_{i}}{t}$$

*S*_i*-*1_ – ulcer area of the previous measurement (1 week earlier) [cm^2^] *S*_i_–ulcer area on a given measurement day [cm^2^] *t*–time (in days) between measurements [day].

#### 2.2.2 Dressings characteristics

In patients from experimental group the cellulose membrane Bioprocess® (Biofil, Productos Biotechnologicos A.A. Curitiba, Brasil) was used. This membranę is formed by a microfibrilar net of homogeneous cellulose, produced by bacterial biosynthesis (*Acetobacter* sp.). It resembles human skin, is semi-transparent and has an average thickness of 0.05 mm, as well as selective permeability to water vapour and substances dissolved in it. Bacteria are unable to penetrate an intact membrane. BC is a nanomaterial, as the thickness of its fibres does not exceed 130 nm, and the nanofibrils that form them have a cross-section of 5–10 nm × 30–35 nm. A membrane formed from bacterial cellulose is selectively permeable to gases such as O_2_, N_2_ or CO_2_ and water and substances dissolved in it (e.g., glucose or NaCl) and impermeable to microorganisms.

The R version of the Kedem-Katchalsky-Peusner (K-K-P) formalism was used to evaluate the transport properties of the BC membrane (Peusner, 1985; Ślęzak-Prochazka et al., 2022):

$$\left[\begin{array}{c} \Delta P-\Delta \pi \\ \frac{\Delta \pi }{C} \end{array}\right]=\left[R\right]\left[\begin{array}{c} J_{v}\\ J_{s} \end{array}\right]=\left[\begin{array}{cc} R_{11} & R_{12}\\ R_{21} & R_{22} \end{array}\right]\left[\begin{array}{c} J_{v}\\ J_{s} \end{array}\right]$$

Where $R_{11}=\frac{\omega +L_{p}C{\left(1-\sigma \right)}^{2}}{L_{p}\omega }$ , $R_{12}=R_{21}=-\frac{1}{\omega }\left(1-\sigma \right)$ , $R_{22}=\frac{1}{C\omega }$ , $C=\left(C_{h}‐C_{l}\right){\left[\text{In}\left({C_{l}}^{‐1}\right)\right]}^{‐1}$ , $C_{h}$ and $C_{l}$ – solution concentrations ( $C_{h}$ > $C_{l}$ ), ΔP – hydraulic pressure difference, Δπ – osmotic pressure difference, $J_{v}$ – volume flux, $J_{s}$ – solute flux.

The coefficients $R_{11}$ , $R_{12}$ , $R_{21}$ and $R_{22}$ appearing in equation above act as resistance coefficients. In order to calculate them, the coefficients of hydraulic permeability ( $L_{p}$ ), reflection ( σ ) and diffusion permeability ( ω ) must first be determined experimentally in accordance with the procedure described in (Ślęzak-Prochazka et al., 2022). These coefficients act as proportionality coefficients in the classic Kedem-Katchalsky equations. However, their role in the assessment of membrane transport cannot be overestimated. For example, the reflection coefficient ( σ ) is a measure of membrane selectivity. Its values are in the range 0 ≤ σ ≤ 1. The membrane is non-selective if σ = 0, selective if 0 < σ < 1. If σ = 1, the membrane is semi-permeable. Table 3 summarises the numerical values of these coefficients for aqueous glucose solutions and aqueous NaCl solutions

**TABLE 3:** Values of coefficients: hydraulic permeability (*L_p_*), reflection (*σ*) and diffusion permeability (*ω*) for the Bioprocess membrane and aqueous glucose and NaCl solutions.

Solution	$L_{p}$ × 10^11^ m^3^ N^−1^s^-1^	σ × 10^2^	ω × 10^10^ mol N^−1^s^-1^
Glucose NaCl	6.5 ± 0.3 6.5 ± 0.3	0.43 ± 0.08 0.36 ± 0.07	10.7 ± 0.5 17.1 ± 0.6

Table 4 shows that the BC membrane has good hydraulic and diffusion permeability properties for aqueous solutions of glucose, ethanol and NaCl. It also has certain selectivity characteristics, as evidenced by the non-zero value of the A coefficient.

**TABLE 4:** Values of resistance coefficients $R_{11}$ and $R_{22}$ for the BC membrane and aqueous glucose and NaCl solutions.

​	$R_{11}$ × 10^11^ Ns m^-3^		$R_{22}$ × 10^10^ N m^-3^mol^-2^	
$\bar{C\;}$ [mol m^-3^]	Glucose	NaCl	Glucose	NaCl
2.79 5.41 7.68 9.77 11.75 13.66 15.51 17.32 19.08 20.81	0.18 0.20 0.22 0.24 0.26 0.28 0.30 0.31 0.34 0.35	0.17 0.18 0.20 0.21 0.22 0.23 0.24 0.25 0.26 0.27	0.035 0.017 0.012 0.009 0.008 0.007 0.006 0.005 0.005 0.004	0.021 0.011 0.008 0.006 0.005 0.004 0.004 0.003 0.003 0.002

In turn, the values of the coefficients $R_{12}$ and $R_{21}$ are equal. This means that Onsager’s reciprocity rule is satisfied for these coefficients. For aqueous glucose solutions, $R_{12}$ = $R_{21}$ = −0.093 × 10^10^ Ns mol^-1^ and for NaCl $R_{12}$ = $R_{21}$ = −0.058 × 10^10^ Ns mol^-1^.

#### 2.2.3 Wound care procedure

In the beginning, the ulcers were thoroughly debrided, with the removal of eschar, fibrin and, when necessary, necrotic tissue. Necrotic areas were eliminated either surgically or through the application of enzyme-based ointments combined with antiseptic irrigation.

The ulcers in experimental group were rinsed with physiological sodium chloride solution and cellulose membrane was placed directly to the wound surface about 1.0 cm beyond the edges of the wound. The membrane was then covered with a sterile secondary dressing (gauze pads) and secured with a non-elastic compression bandage. For the proper measurement of compression pressure, the Kikuhime device pressure sensor was used and a pressure of 25–35 mmHg was applied. The dressing was changed once a week (every 7 days).

In contrast, patients in the control group were treated with an antimicrobial calcium alginate dressing containing silver (Suprasorb A+ Ag, Lohmann & Rauscher, Pabianice, Poland), which was applied directly to the wound surface following irrigation of the wound with physiological saline. Subsequently, as in experimental group, gauze pads were placed over the ulcer, the limb was wrapped with a non-elastic supportive compression bandage. A compression pressure of 25–35 mmHg was precisely maintained using the Kikuhime device. The Suprasorb A+ Ag dressing was replaced daily until complete ulcer healing was achieved.

Before removal from the ulcer, dressings were moistened with physiological sodium chloride solution. After 30–60 s, once the dressing has softened, it was be gently removed. Removal was performed using forceps or sterile gauze, proceeding slowly and without forceful traction. The bacterial cellulose membrane occurred to be easier to remove and caused less adherence to the wound surface compared to the alginate dressing.

Throughout the study, none of the patients in either group discontinued or refused the proposed treatment.

### 2.3 Statistical analysis

Data was collected in an Excel spreadsheet and then exported to STATISTICA 13.3 software (StatSoft Polska, Krakow, Poland), where statistical calculations were performed.

The percentage changes in the reduction of the ulcer area in individual weeks and the healing rate in each week–healing index per day (cm^2^/day) - were calculated. Non-parametric tests were used for comparisons, as the data in individual groups did not show signs of normality (checked with the Kolmogorov-Smirnov test). Values for the two groups were compared using the Mann-Whitney U test. The chi-square test was used for qualitative comparisons. A level of p < 0.05 was considered statistically significant.

## 3 Results

### 3.1 Complete healing time

In all 50 patients in experimental group, the ulcers healed successfully (i.e., completely) within the first 14 weeks of treatment by topical cellulose membranous Bioprocess® application. Ulcer healing was also observed in all the cases in control group (antimicrobial calcium alginate wound dressing + Ag). However, within the first 14 weeks of treatment, complete healing was found only in 40 out of 50 patients in this group and the time up to the complete healing of the last ten cases was significantly longer — 16 weeks (Table 5). The maximum time of the ulcer healing in experimental group was significantly shorter (98 days) than in control group (112 days); p < 0.05.

**TABLE 5:** The number of new patients with completely healed ulcers depending on the duration of treatment.

Group	Duration of treatment (weeks)															
	1	2	3	4	5	6	7	8	9	10	11	12	13	14	15	16
Experimental (Bioprocess®)	​	​	​	8	10	10	​	10	​	​	8	​	​	4	​	​
Control (Suprasorb A+ Ag)	​	​	​	6	​	6	​	7	​	7	5	4	3	2	4	6

### 3.2 Cost-effectiveness

Cost analysis indicated that although the unit price of a bacterial cellulose dressing (12.00 PLN) was higher than that of a silver-containing alginate dressing (6.36 PLN), the overall treatment cost over a 4-week period was substantially lower for bacterial cellulose (48.00 PLN vs. 178.08 PLN). This difference resulted from less frequent dressing changes (once weekly vs. daily). Furthermore, the use of bacterial cellulose significantly reduced overall healthcare system costs, as nursing time was approximately sevenfold lower.

The observed difference in dressing change frequency reflects the distinct functional properties of the materials used. The alginate dressing requires daily replacement due to its high absorptive capacity combined with rapid saturation in moderately to heavily exuding wounds (notably, the wounds included in our study were characterized by moderate to heavy exudate). Frequent replacement is therefore necessary to maintain effectiveness, prevent periwound maceration, and support antimicrobial activity associated with silver ions. In contrast, bacterial cellulose, owing to its nanofibrillar structure and high water content, enables both fluid absorption and retention, thereby maintaining a stable moist wound environment over prolonged periods. Its structural integrity and close adherence to the wound bed reduce the need for frequent intervention, allowing for once-weekly changes without compromising local wound conditions.

These material-specific properties not only justify the applied treatment protocols but also directly contribute to improved cost-effectiveness by reducing the number of dressing changes and associated nursing time while preserving optimal conditions for wound healing.

### 3.3 Ulceration area reduction

**FIGURE 1:**
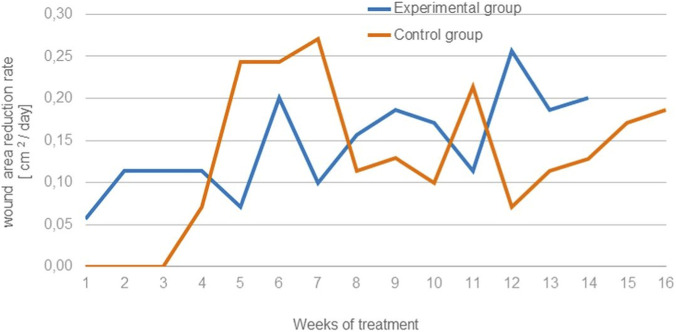
Changes in ulceration area in subsequent weeks of treatment; differences are not statistically significant, but overall wound area reduction is faster in experimental group, especially in the beginning and in the end of healing process.

**FIGURE 2:**
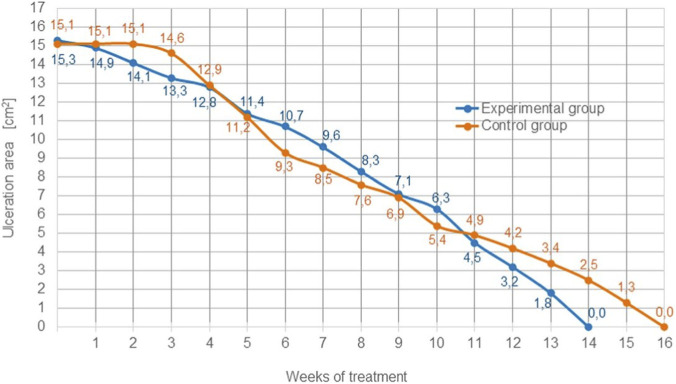
The rate of wound area reduction depending on the treatment time; differences are not statistically significant, but overall wound area reduction rate is more stable in experimental group.

In experimental group, fifty patients (baseline mean ulcer area, 15.3 cm^2^) were treated with the Bioprocess® cellulose membrane. After 7 days, the mean area decreased by 0.4 cm^2^ (v_1_ = 0.057 cm^2^/day). After a further 21 days, the rate of reduction was 0.114 cm^2^/day and the mean area was 12.8 cm^2^. During the subsequent 7 days, ulcers in seven women and three men healed; the mean area decreased by 1.4 cm^2^ and v_1_ = 0,20 cm^2^/day. In the next 7 days, complete healing occurred in 10 additional patients; the mean area was 10.7 cm^2^ and the rate of reduction was 0.10 cm^2^/day. After a further 7 days, the mean area decreased by 1,1 cm^2^ (v_1_ = 0.157 cm^2^/day). One week later, ulcers in another 10 patients had healed (v_1_ = 0.186 cm^2^/day), and the mean area fell to 7,1 cm^2^. After 14 days of treatment, the mean area decreased by 2,6 cm^2^; the reported rates of reduction were 0.171 and 0.114 cm^2^/day. After 11 weeks of treatment, ulcers in four women and four men had healed; the mean area decreased to 4,5 cm^2^ and v_1_ reached its maximum of 0.257 cm^2^/day. Over the next 2 weeks, the mean area declined to 1,8 cm^2^, with v_1_ values of 0.186 and 0,20 cm^2^/day. Complete healing of the last four patients occurred during the following 7 days, with v_1_ = 0.257 cm^2^/day (Figures 1, 2).

In control group fifty patients (baseline mean ulcer area, 15,1 cm^2^) received standard treatment. During the first 3 weeks, the mean ulcer area did not change (15.1 cm^2^). After a further 7 days, the mean area decreased by 0.5 cm^2^ (v_2_ = 0.071 cm^2^/day); ulcers in three women and three men healed completely. Over the next 2 weeks, the rate of area reduction was 0.243 cm^2^/day and the mean area reached 9,3 cm^2^; ulcers in four women and two men healed. During the subsequent 7 days, the mean area decreased by 1,2 cm^2^ (reported v_2_ = 0.271 cm^2^/day). After another 7 days, the rate of reduction was 0.114 cm^2^/day with a mean area of 7,6 cm^2^; seven additional patients healed. One week later, v_2_ was 0.129 cm^2^/day and the mean area 6,9 cm^2^. After a further 7 days, the mean area decreased by 1,5 cm^2^ (v_2_ = 0,10 cm^2^/day). After 11 weeks of dressing use, the mean ulcer area had decreased by 10,2 cm^2^ relative to baseline; during this period, the highest observed rate of reduction was 0.214 cm^2^/day, and ulcers in five women healed. After an additional 7 days, v_2_ was 0.071 cm^2^/day with a mean area of 4,2 cm^2^; four more patients healed. In the next 7 days, the mean area decreased by 0,8 cm^2^ (v_2_ = 0.114 cm^2^/day) and three patients healed. After a further week, v_2_ was 0.128 cm^2^/day with a mean area of 2,5 cm^2^; two patients healed. In the subsequent 7 days, the mean area decreased by 1,2 cm^2^ (v_2_ = 0.171 cm^2^/day) and ulcers in four women healed. In the final week, the remaining six patients achieved complete healing (v_2_ = 0.186 cm^2^/day) (Figures 1, 2).

The findings indicate that treatment with the Bioprocess® cellulose membrane was associated with a more rapid and consistent reduction in ulcer area compared with standard therapy. In the experimental group, a gradual yet steady acceleration in healing rate was observed over the course of treatment, culminating in a maximum reduction velocity (v_1_) of 0.257 cm^2^/day and complete ulcer closure in all patients. Notably, a significant decrease in ulcer area occurred as early as the first month of treatment, with progressive increases in healing velocity during later stages.

In contrast, patients in the control group who received standard treatment exhibited delayed onset of healing, with no measurable area reduction during the first 3 weeks. Subsequent improvements were slower and less uniform, with a maximum reduction rate (v_2_) of 0.214 cm^2^/day, achieved later in the treatment period. Although complete healing was ultimately achieved in all cases, the overall healing dynamics were less favorable than those observed in the experimental cohort.

**FIGURE 3:**
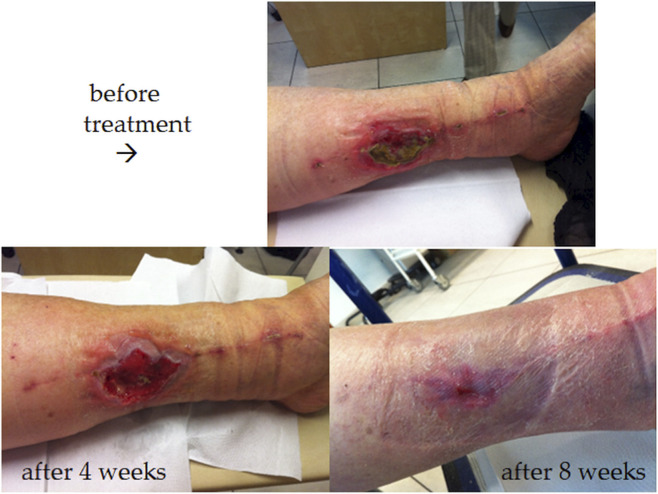
Ulcer healing process (during BC treatment) of 65-year-old woman with a VLU, that had been unsuccessfully treated for 20 months.

**FIGURE 4:**
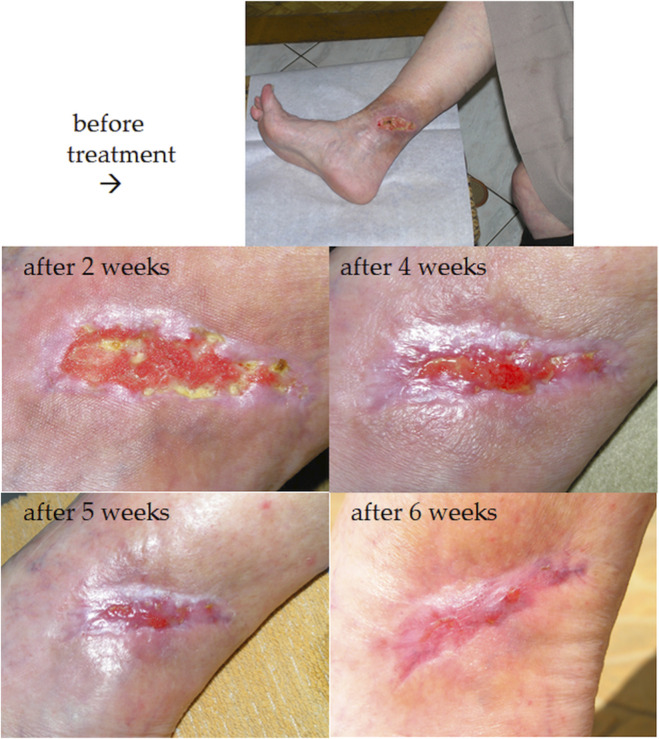
Ulcer healing process (during BC treatment) of a 68-year-old woman with a VLU, that had been unsuccessfully treated for 24 months.

Sample clinical images showing the wound healing process over time in two patients from the experimental group are presented in Figures 3, 4.

## 4 Discussion

The treatment of chronic and non-healing wounds represents a major challenge for modern healthcare systems. This large patient population primarily includes individuals suffering from leg ulcers, pressure sores, and diabetic foot ulcers. The duration of wound healing is directly correlated with overall treatment costs. Prolonged healing times result in extended hospital stays and increased consumption of medical materials, consequently driving up healthcare expenditures. Accelerating the wound-healing process shortens treatment duration and reduces costs by decreasing the frequency of dressing changes.

Technological progress has led to the development of numerous synthetic wound dressings based on polymeric materials. Although traditional dressings such as gauze and cotton are still commonly used due to their low cost and accessibility, they are often associated with delayed wound healing. In this context, evaluating the long-term application of bacterial cellulose (BC)-based dressings may provide both clinical and economic benefits.

Calcium alginate dressings impregnated with silver ions (used in the control group) are widely used in the management of venous ulcers because of their high exudate absorption capacity and broad-spectrum antimicrobial activity (O’Meara et al., 2015; Zhao et al., 2020). However, recent meta-analyses have questioned whether silver confers any significant advantage in accelerating wound closure in chronic wounds, suggesting that its benefit may be limited to the early phase of healing or to wounds with heavy bioburden (Liang et al., 2024). In contrast, BC dressings provide a moist, oxygen-permeable environment that maintains optimal hydration while limiting microbial penetration, without the potential cytotoxicity sometimes associated with silver ions. The results of the present study therefore support the hypothesis that a non-cytotoxic, hydrophilic BC membrane can achieve comparable or superior healing outcomes to silver-based alginates in chronic venous ulcers.

BC fulfills the requirements of an advanced wound dressing due to its excellent physicochemical and biological properties. Its flexibility enables application to wounds with irregular margins, while its semitransparency facilitates continuous observation of the wound bed. *In vitro* studies have shown that BC dressings promote cell adhesion, growth, and proliferation of various cell types, including fibroblasts, keratinocytes, and endothelial cells (Fu et al., 2013).

Interestingly, BC influences all phases of wound healing—namely inflammation, proliferation, and remodeling. Its three-dimensional network structure effectively prevents microbial invasion while allowing adequate gas and fluid exchange during the healing process. Furthermore, BC can serve as a carrier for bioactive substances such as collagen, chitosan, graphene, silver, and polyhexamethylene biguanide (PHMB), as well as for antibiotics, thereby enhancing the healing process (Mensah et al., 2021; Szkiler et al., 2024).

Comparative characteristics of Bioprocess® (Bacterial Cellulose) and Suprasorb A+ Ag (alginate with silver) wound dressings is presented in Table 6.

**TABLE 6:** Comparison of physicochemical and clinical properties of Bioprocess^®^ and Suprasorb A+ Ag wound dressings.

Parameter	Bioprocess® (bacterial cellulose)	Suprasorb A+ Ag (alginate + silver)
Dressing type	Bioactive/“artificial skin”	Absorptive + antibacterial
Mechanism of action	Maintenance of a moist environment; promotion of a healing microenvironment	Exudate absorption + release of Ag^+^ ions
Exudate level	Moderate	High/very high
Infection control	No antibacterial activity	Strong antibacterial activity
Pain	↓ Pain (protection of nerve endings)	Neutral
Dressing change frequency	Every 7–10 days	Every 1–3 days
Adherence	Very good (bioadhesion)	Non-adherent (gel-forming)
Cytotoxicity	None	Possible (Ag effect on keratinocytes)
Biofilm	No activity	Active against biofilm
Wound healing	Direct support	Indirect support (via infection control)

Indeed, our results prove that the Bioprocess® cellulose membrane accelerates wound healing, promotes earlier onset of tissue regeneration, and sustains a higher rate of ulcer area reduction compared to conventional therapy. Complete epithelialization was achieved within 14 weeks in the BC group, whereas the control group required approximately 16 weeks (112 days) of treatment (Table 3). The data support its potential clinical utility as an effective advanced dressing for the management of chronic ulcers.

The superior healing observed in the BC group can be attributed to the distinct physicochemical and biological features of bacterial cellulose. BC is characterized by a highly pure nanofibrous structure composed of β(1→4)-linked glucose chains, with an exceptionally high density of hydroxyl groups that confer strong hydrophilicity and water retention capacity—up to 60–700 times its dry weight (Portela et al., 2019). This property maintains a consistently moist microenvironment favorable for keratinocyte and fibroblast proliferation while preventing desiccation of the wound bed. Furthermore, the interconnected porous network of BC allows gas exchange (O_2_, CO_2_) but acts as a barrier to microorganisms (Girard et al., 2024). This nanofibrillar three-dimensional network of BC closely mimics the structure of the native extracellular matrix (ECM), providing a highly hydrated, oxygen-permeable, and mechanically stable scaffold that facilitates cell migration and proliferation (Utoiu et al., 2024; Dogaru et al., 2025). It also allows the infiltration of fibroblasts and endothelial cells, supporting angiogenesis and granulation tissue formation—key processes in the repair of chronic venous ulcers (He et al., 2021; Han et al., 2023). In preclinical studies, BC scaffolds have been shown to enhance capillary formation and upregulate vascular endothelial growth factor (VEGF) expression, thereby accelerating neovascularization (Han et al., 2023; Dogaru et al., 2025). Furthermore, BC maintains a moist wound environment that promotes keratinocyte migration and re-epithelialization while preventing desiccation or secondary trauma during dressing changes (Silva et al., 2021; Sung et al., 2025)

The accelerated wound closure observed in our trial is therefore consistent with the known cellular mechanisms of BC-mediated tissue repair. Abovementioned physicochemical characteristics likely explain the observed continuous reduction in ulcer area and shorter healing time in the BC-treated group. Similar mechanisms have been proposed in other clinical applications of BC-based dressings, including partial-thickness burns and donor-site wounds (He et al., 2021).

The present study is a kind of continuation of the experiment of the authors who compared the efficacy of a cellulose membrane dressing (Bioprocess®) in the treatment of hard-to-heal VLUs with that of a traditional hydrocolloid dressing (Unna’s boot). Patients treated with the cellulose membrane (mean ulcer area 28.9 cm^2^) achieved complete healing in 15 cases within 8 weeks, while the remaining ulcers closed over the following 6 weeks. In contrast, among patients treated with Unna’s boot (mean ulcer area 28.7 cm^2^), complete epithelialization occurred in only four cases after 8 weeks, and the remaining ulcers required up to 20 weeks for full closure (Kucharzewski et al., 2003).

Other authors also confirm superiority of BC over other traditional dressings. Alvarez et al. (2004) reported that BC dressings (XCell) promoted faster autolytic debridement and granulation than standard non-adherent dressings in a cohort of 24 patients with venous leg ulcers. Their study showed a significant reduction in necrotic tissue (*p* = 00,094), earlier formation of granulation tissue (>75% at 43 vs. 71 days), and higher wound area reduction at both 6 and 12 weeks. Consistent with those results, our study confirms the enhanced healing kinetics of BC dressings, with complete closure achieved earlier than with the alginate-silver control, suggesting that BC provides an optimal moist environment and supports tissue regeneration.

Similarly, Silva et al. (2021) demonstrated faster wound area reduction and decreased pain in patients treated with BC dressings compared with cellulose acetate mesh dressings impregnated with essential fatty acids. Our findings corroborate these observations, as patients treated with BC reported improved comfort during dressing changes and showed earlier wound closure, emphasizing BC’s biocompatibility and non-adherent nature.

The results of Almeida et al. (2014), Cavalcanti et al. (2017) confirmed the safety and non-toxicity of BC dressings, showing no skin irritation and suggesting BC-induced tissue remodeling after prolonged use. The absence of local complications in our cohort aligns with these reports, reinforcing the biocompatibility and clinical safety of BC in long-term wound management. Colenci et al. (2019) further demonstrated that a BC biomembrane (Nanoskin®) used in conjunction with compression therapy was comparable in efficacy to collagenase-based treatment in venous ulcers, with earlier healing observed in the BC group (*p* = 0.02). Our findings extend these results, showing that BC not only performs comparably to enzyme-based dressings but also surpasses silver alginate in overall healing efficiency.

Taken together, the current and previous studies confirm that BC-based dressings provide a favorable environment for wound repair through superior moisture balance, biocompatibility, and promotion of tissue granulation and re-epithelialization. From a translational perspective, bacterial cellulose demonstrates high potential for integration into routine clinical practice, due to their ability to promote healing (even in the presence of inflammation), reduce pain, and lower treatment costs. Advances in biotechnology now allow large-scale, sterile production of BC membranes with tunable thickness and surface chemistry, enabling incorporation of bioactive compounds such as growth factors, antimicrobials, or metallic ions (Han et al., 2023; Dogaru et al., 2025). This opens pathways toward next-generation “smart” dressings capable of simultaneously promoting regeneration and preventing infection. Furthermore, the ability to customize BC membranes into specific shapes and sizes makes them promising for complex wounds and potentially reduces the overall cost of VLU treatment. The present study provides valuable clinical confirmation of BC’s regenerative efficacy in chronic venous ulcers, thus bridging the gap between laboratory research and therapeutic application. The observed reduction in healing time in our study further underscores the potential of BC as a cost-effective and clinically efficient alternative to conventional dressings.

The use of planimetric measurements every 7 days provided objective and reproducible quantitative data on wound area reduction, consistent with validated digital planimetry methods described in the literature (Jørgensen et al., 2016; Ibraheem et al., 2023). However, several limitations should be acknowledged. Firstly, although the sample size was sufficient to demonstrate statistically significant differences between the treatment groups, a larger multicenter study would be required to confirm the generalizability of the findings. Secondly, the observation period was limited to the time required for complete ulcer healing, without long-term follow-up to assess recurrence rates or durability of the therapeutic effect. Additionally, potential confounding factors such as patients’ comorbidities, nutritional status, and adherence to compression therapy were not fully controlled. Finally, the study compared only one type of cellulose-based dressing with a single control dressing, which restricts broader comparisons with other advanced wound care modalities.

From a clinical standpoint, the results suggest that BC membranes represent a promising, biocompatible alternative to traditional alginate dressings for chronic venous ulcers, offering comparable antimicrobial protection through physical rather than chemical means. Indeed, BC has recently emerged as a promising biomaterial in regenerative medicine because of its exceptional physicochemical properties, such as high purity, moisture retention, and structural similarity to the ECM.

However, future research should focus on prospective, randomized controlled trials with larger and more diverse patient populations to validate the observed benefits of bacterial cellulose dressings in venous leg ulcer management. Long-term follow-up studies are warranted to evaluate the recurrence rate, quality of life improvements, and cost-effectiveness of this therapeutic approach. Furthermore, mechanistic investigations at the cellular and molecular levels could provide deeper insight into the biological interactions between bacterial cellulose and the wound microenvironment. Comparative studies including other advanced wound dressings and combination therapies may also help optimize treatment protocols and identify patient subgroups most likely to benefit from cellulose-based interventions.

### 4.1 Conclusion

The results of this study demonstrate that BC membranes significantly enhance the healing process of chronic venous leg ulcers compared with conventional silver-containing alginate dressings. Patients treated with the cellulose-based material exhibited a faster reduction in ulcer area and a shorter overall healing time. These findings highlight the advantageous physicochemical and biological properties of bacterial cellulose, which support effective wound repair and create a favorable healing environment. Given its biocompatibility, non-toxicity, and capacity to maintain optimal moisture balance, bacterial cellulose represents a promising alternative to traditional dressings in chronic wound management. Further large-scale, prospective studies are warranted to confirm these results, evaluate long-term outcomes, and explore the cost-effectiveness and broader clinical applications of cellulose-based biomaterials in the treatment of venous leg ulcers. Future investigations should also focus on optimizing BC dressings with integrated bioactive agents or antimicrobial compounds to further enhance their therapeutic performance in chronic wound management.

## Data Availability

The raw data supporting the conclusions of this article will be made available by the authors, without undue reservation.
